# Malignant melanoma of the stomach presenting in a woman: a case report

**DOI:** 10.1186/1752-1947-5-94

**Published:** 2011-03-09

**Authors:** Vedat Goral, Feyzullah Ucmak, Serdar Yildirim, Sezgin Barutcu, Serdar İleri, İlknur Aslan, Huseyin Buyukbayram

**Affiliations:** 1 Department of Gastroenterology, Dicle University School of Medicine, 21280 Diyarbakir, Turkey; 2 Department of Internal Medicine, Dicle University School of Medicine, 21280 Diyarbakir, Turkey; 3 Department of Family Medicine, Dicle University School of Medicine, 21280 Diyarbakir, Turkey; 4 Department of Pathology, Dicle University School of Medicine 21280 Diyarbakir, Turkey

## Abstract

**Introduction:**

Malignant melanoma is reported to metastasize to all organs of the human body. Although it is common for it to metastasize to the gastrointestinal tract, a melanoma located primarily in the gastric mucosa is an uncommon tumor. Gastrointestinal metastases are rarely diagnosed before death with radiological and endoscopic techniques.

**Case presentation:**

In this case report the clinical course and treatment of a woman with melanoma of the stomach, without any other detectable primary lesion, is presented and discussed. A 55-year-old Turkish woman presented to our clinic with complaints of muscle pain and bone pain in the left side of her chest. During an upper gastrointestinal system endoscopy, dark cherry-colored, light elevated, round-shaped lesions were taken from her gastric fundus and from the first part of her duodenum. Biopsies from these samples were determined to be malignant melanoma by the pathologist.

**Conclusion:**

Metastatic malignant melanoma cases should be examined through endoscopy for gastrointestinal metastases.

## Introduction

Malignant melanoma is reported to metastasize to all organs of the human body [1-4]. Although it is common for it to metastasize to the gastrointestinal tract (GIT), a melanoma located primarily in the gastric mucosa is an uncommon tumor [5,6]. Gastrointestinal metastases are rarely diagnosed before death, using radiological and endoscopic techniques [7-9]. Also, GIT metastases can appear in various morphological forms, and therefore immunohistochemistry is often useful in distinguishing between a malignant melanoma and other malignancies. The median survival time for melanoma patients presenting with gastrointestinal invasion is less than one year [2]. The prolonged survival time reported in a few patients with gastrointestinal metastases is associated with aggressive surgical treatment, adjuvant chemotherapy and immunotherapy. The high mortality rate observed in these patients is associated with multiple metastases to other organs, such as lungs, liver, pancreas, spleen, endocrine glands, and brain [6]. In this case report, the clinical course and treatment of a woman with melanoma of the stomach, without any other detectable primary lesion, is presented and discussed.

## Case presentation

A 55-year-old Turkish woman presented to our clinic with complaints of muscle pain and bone pain in the left side of her chest. She had a diagnosis of malignant melanoma leading to amputation of her left great toe six years ago. This lesion was a primary focus of malignant melanoma. She did not have any metastases. One month prior to this event, our patient felt pain under her left breast; coronary angiography results were normal. The patient attended our Physical Therapy clinic, where no significant abnormalities were found during the examination. She was then referred to our Gastroenterology clinic with a diagnosis of liver abnormality. Initial tests in our clinic revealed she had a white blood cell count of 11.1 k/ul, a hematocrit of 31.4, a platelet count of 437.0 k/uL, positive result for hepatitis B surface antigen, a hepatitis B virus DNA level of 2.17 × 10^3^IU/L, aspartate transaminase levels of 8IU/L, alanine transaminase levels of 70IU/L, alkaline phosphatase levels of 275IU/L, lactate dehydrogenase levels of 1206IU/L and gamma-glutamyltransferase levels of 221IU/L. Blood urea, creatinine, amylase, sodium, potassium, chlorine and calcium ion levels, total bilirubin, thyroid-stimulating hormone, triiodothyronine, thyroxin, free triiodothyronine and free thyroxin levels were all normal. Further tests showed subsequent levels of cancer antigen (CA) 125 to be 68.17U/ml (normal range, 1-35U/mL), CA 15-3 to be 23.08U/mL (normal level < 25U/mL), CA 19-9 to be 23.62U/mL (normal level < 40U/mL), ferritin to be 534.8 ng/mL and folate to be 8.67 ng/mL.

**Figure 1:**
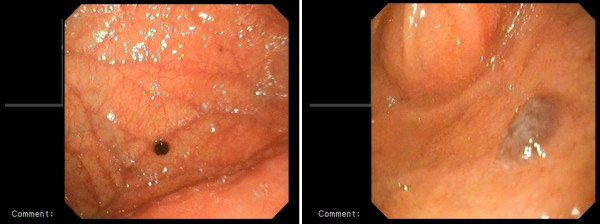
**Endoscopic images of gastric metastasis of the malignant melanoma**.

**Figure 2:**
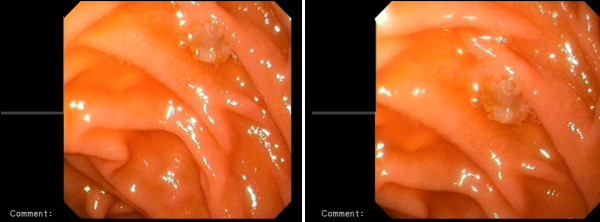
Endoscopic images of duodenal metastasis of the malignant melanoma.

**Figure 3:**
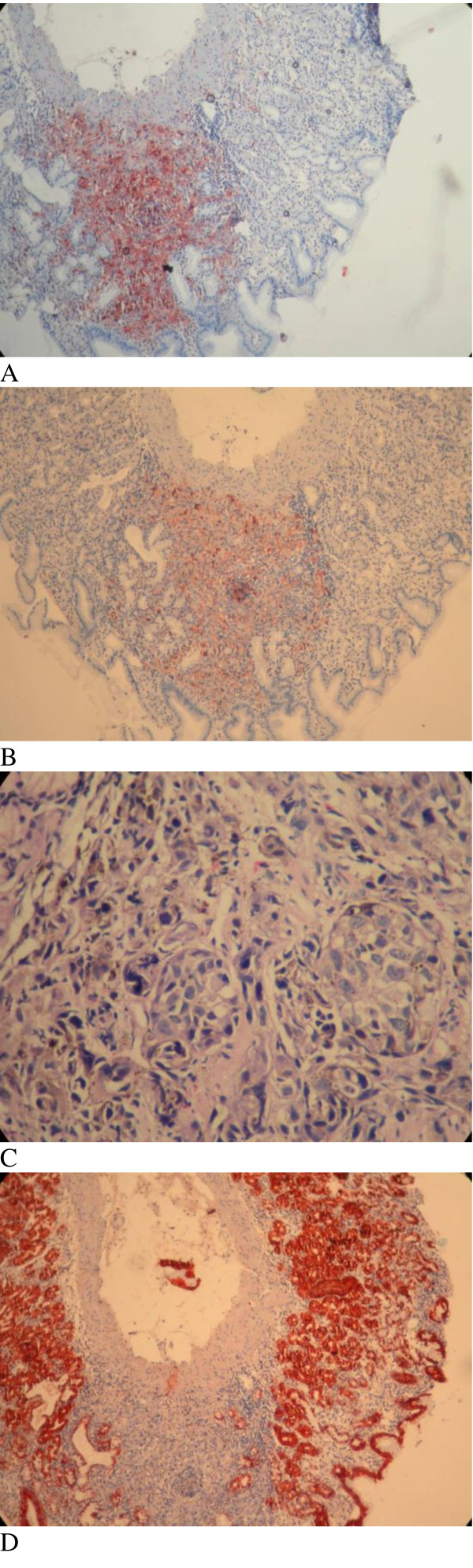
**Histopathologic image of gastric metastasis of the malignant melanoma**. Immunohistochemical staining was performed for S-100 (A), Melan-A (B), H&E (C), and CK (D). Melan-A (MART-1): A new monoclonal antibody for malignant melanoma diagnosis.

Total abdominal-pelvic ultrasonography indicated a growth in the total size of her liver (craniocaudal diameter 182 mm), with many hypoechoic lesions (metastases), the largest with a diameter of 23 mm at the porta hepatis, paraaortic, and in the peripancreatic region with the largest having a diameter 20 mm. These hypoechoic lesions are perhaps suggestive of metastatic lymphadenopathy. Other intraabdominal organs were normal. Computed tomography (CT) of her thorax showed enlarged right supraclavicular, upper mediastinal, paratracheal, subcarinal, right hilar, left axillary and peridiaphragmatic (largest with a diameter of 35 mm) lymph glands. Several nodes were observed on both lungs with the largest ones having diameters of 12 mm on her right lung and 11 mm on her left lung. Bone structures in the observed region showed lithic lesions (metastasis). Given that our patient had a history of malignant melanoma leading to amputation of her left great toe, upper and lower GIS endoscopy were administered. During upper GIS endoscopy, dark cherry-colored, light elevated, round-shaped lesions were taken from her gastric fundus and from the first part of her duodenum (Figures 1 and 2). Biopsies from these samples were determined to be malignant melanoma by the pathologist (Figure 3). A colonoscopy revealed a polyp; a biopsy was taken and evaluated to be a tubular adenoma.

Current findings for our patient were assessed to indicate a malignant melanoma with metastasis to her stomach, liver, lungs and bones. Stomach metastasis due to a malignant melanoma is very rare, and such metastases are hardly ever reported among gastric metastases. This case is the first gastric and duodenal metastases observed in our clinic due to malignant melanoma. Our patient was referred to our oncology clinic after the diagnosis, for chemotherapy.

## Discussion

Malignant melanoma is known to metastasize to different organs of the human body with an unusual predilection for the gastrointestinal tract. Gastrointestinal invasion is a rare condition and is often associated with the invasion of other visceral organs [6]. Malignant melanoma of the GIT is a rare entity among intestinal neoplasms. Primary intestinal melanoma is difficult to differentiate from metastatic melanoma, especially given that the primary cutaneous lesion has the potential to regress and disappear. In addition, melanoma by itself is a great mimicker of other neoplastic conditions and may create a major diagnostic challenge when presenting at an intraabdominal location. The mean survival time of these patients is consistently less than one year. The exact clinical incidence of gastrointestinal melanoma cannot be determined from any large series, but the stomach, after the small bowel, is the second most common site involved [6]. Autopsy frequently reveals gastrointestinal involvement in patients that have died from melanoma, however little evidence emerges in antemortem diagnosis and, even then, usually only in connection with emergency situations such as obstructions, bleeding or perforation. The frequently asymptomatic character of gastrointestinal melanoma explains why it largely eludes detection. Symptoms include mainly gastrointestinal bleeding, abdominal pain, anorexia, nausea and vomiting, weight loss, progressive dysplasia, obstruction, and occasionally acute perforation. Melena in a melanoma patient seems to be a primary symptom for gastrointestinal metastasis, even in the absence of other symptoms [10]. In our case our patient never experienced melena. In the majority of the cases reported, the gastric involvement was a manifestation of terminal metastasis. It has been reported that almost all the areas of the human body can be affected by melanoma metastases.

Many of the previous reports on the gastric spread were based on the radiological features of the metastases. Recently, however, endoscopy has been shown to be a more reliable diagnostic tool [7-9]. It permits exact morphological evaluation and direct biopsy for pathological diagnosis. Moreover, by endoscopic follow-up it is possible to monitor the course of metastases and to evaluate the results of treatment. The endoscopic classification of the gastric metastases comprises three main morphological types. Firstly there are melanotic nodules, often ulcerated at the tip, which are the most frequently observed endoscopic feature. Secondly are submucosal tumor masses, melanotic or not, which are elevated and ulcerated at the apex. This is the typical aspect of "bull's eye" lesions. The third morphological type is mass lesions, with varying incidence of necrosis and melanosis. Additionally, gastric metastases may appear even as a simple ulcer [6]. Concerning the anatomical site of the gastric metastases, the majority of them are reported to occur in the body and the fundus, most often at the greater curvature with lesser curvature lesions being uncommon. In our patient, the endoscopic picture of the gastric lesion showed it to be melanotic at her gastric fundus and the first part of her duodenum. The pathological evaluation could confirm the metastatic nature of the melanoma lesion. GIT metastases can appear in various morphological forms, and therefore immunohistochemistry is often useful in distinguishing between a malignant melanoma and other malignancies (11,12].

Although surgical treatment has been attempted in some melanoma patients with gastrointestinal metastases, surgery seems to be of limited practical value and should be performed only in carefully selected patients and in patients with complications. The poor general condition of our patient by the time of the diagnosis, complicated with other organ (liver, bone and lungs) metastases, did not allow any surgical treatment [6].

## Conclusion

Metastatic melanoma in various areas, from an unknown primary lesion, is well documented in the literature [1-12]. The stomach, after the small bowel, is the second most common site involved. The primary origin of a melanoma in the stomach is extremely unlikely and can be accepted only if the absence of any other primary lesion is confirmed. Endoscopy has been shown to be the most reliable form of examination for the diagnosis of gastric metastases. In addition, gastric invasion is most often associated with the invasion of other organs and the mean survival time of patients presenting with a gastric metastasis is consistently less than one year. Therefore, every metastatic malignant melanoma case should undergo endoscopic examination for gastrointestinal metastases.

## Consent

Written informed consent was obtained from the patient for publication of this case report and any accompanying images. A copy of the written consent is available for review by the Editor-in-Chief of this journal.

## Competing interests

The authors declare that they have no competing interests.

## Authors' contributions

VG and FU diagnosed the lesions endoscopically. SY, SB and SI interpreted the patient data regarding the gastrointestinal and oncologic disease. HB performed the histological examination of the gastric and duodenal lesions of our patient, and was a major contributor in writing the manuscript. All authors read and approved the final manuscript.
